# Epigenomics and Schizophrenia: A Literature Review

**DOI:** 10.1192/bjo.2025.10167

**Published:** 2025-06-20

**Authors:** Nishikant Thorat, Soham Kulkarni

**Affiliations:** 1 Byramjee Jeejeebhoy Government Medical College and Sassoon General Hospitals, Pune, India; 2 Independent Researcher, Pune, India

## Abstract

**Aims:** Schizophrenia is a severe mental illness, characterized by positive, negative, cognitive, affective symptoms with aggression, marked by disrupted structural and functional brain connectivity, as evidenced by neuroimaging, neurophysiological and neuropathological studies. Recent epigenetic research highlights the role of deoxyribonucleic acid (DNA) methylation, histone modifications, and non-coding ribonucleic acid (RNA) amongst others in mediating both genetic predisposition and environmental influences on gene expression as seen in schizophrenia.

**Methods:** A comprehensive search was conducted on PubMed using the keywords “schizophrenia” and “epigenomics”. Information from articles published within the last ten years were selected, including those with free full-text access, including books and documents, clinical trials, meta-analyses, randomized controlled trials, and systematic reviews. Only articles published in the English language were included in the selection process.

**Results:** The brain methylome consists of DNA methylation marks at cytosine and guanosine separated by phosphate group (CpG) sites in the brain’s genome, which regulate gene expression without altering the DNA sequence. This modification influences cellular processes like gene activity, development, and memory. Dysregulation of gene expression, particularly in the prefrontal cortex (PFC), contributes to schizophrenia’s pathophysiology, impacting neurotransmission, myelination, metabolism, and immune signalling.

Histone modifications (acetylation, deacetylation, methylation, phosphorylation) also regulate gene expression, with reduced Histone Deacetylase 2 (HDAC2) expression seen in the dorsolateral PFC of schizophrenia patients. Additionally, microRNAs (miRNAs) and long non-coding RNAs (lncRNAs) are implicated in gene expression dysregulation in schizophrenia, influencing processes like synaptic plasticity and neural differentiation.

Epigenetic changes in peripheral tissues, such as blood and saliva, may serve as biomarkers for schizophrenia.

A comprehensive approach integrates genotyping, epigenotyping, and deep phenotyping to enhance understanding of an individual’s health and treatment responses. Early therapeutic interventions may reverse epigenetic changes, improving outcomes. Incorporating molecular endophenotypes and neuroimaging biomarkers aids in identifying schizophrenia subgroups and enhancing treatment predictions. Omics integration (genomics, transcriptomics, proteomics, metabolomics) increases the precision of schizophrenia risk stratification.

There are various advancements in DNA methylation analysis include high density CpG array system (850,000 sites), whole genome bisulphite sequencing (better resolution but costly), targeted bisulphite sequencing (cost-effective), and emerging single molecule/nanopore sequencing technologies.

**Conclusion:** Current research in schizophrenia reveals interactions between genetic, environmental, and epigenetic factors. While significant advancements have been made in understanding the role of DNA methylation, histone modifications, and non-coding RNAs, further studies with larger sample size and more robust structure along with using multi-omic approach are desirable for understanding the disease pathophysiology and to deliver personalized treatment.

